# Guiding Academic Clinician Educators at Research-Intensive Institutions: a Framework for Chairs, Chiefs, and Mentors

**DOI:** 10.1007/s11606-021-06713-9

**Published:** 2021-04-12

**Authors:** Anna Chang, Brian S. Schwartz, Elizabeth Harleman, Meshell Johnson, Louise C. Walter, Alicia Fernandez

**Affiliations:** 1 Division of Geriatrics, Department of Medicine, University of California San Francisco School of Medicine (UCSF), San Francisco, CA USA; 2 San Francisco Veterans Affairs Health Care System (SFVAHCS), San Francisco, CA USA; 3 Division of Infectious Diseases, Department of Medicine, University of California San Francisco School of Medicine (UCSF), San Francisco, CA USA; 4 Division of Hospital Medicine at Zuckerberg San Francisco General Hospital, Department of Medicine, University of California San Francisco School of Medicine (UCSF), San Francisco, CA USA; 5 Division of Pulmonary Medicine, Department of Medicine, University of California San Francisco School of Medicine (UCSF), San Francisco, CA USA; 6 Division of General Internal Medicine at Zuckerberg San Francisco General Hospital, Department of Medicine, University of California San Francisco School of Medicine (UCSF), San Francisco, CA USA

**Keywords:** clinician educator, academic medicine, mentoring

## Abstract

Department chairs and division chiefs at research-intensive academic medical centers often find mentoring clinician educators challenging. These faculty constitute the majority of academic physicians. Supporting excellent clinician educators is key to ensuring high-quality patient care and developing tomorrow’s physicians. Little has been written for leaders on strategies to advance academic clinician educators’ career success. We present a framework to guide chairs, chiefs, and mentors seeking to address clinician educator retention and satisfaction in academic medical centers.

## The View of Department Chairs and Division Chiefs

Department chairs and division chiefs at research-intensive institutions are challenged by the need to recruit, retain, and support clinician educators.^1, 2^ There is not a single established definition of a clinician educator but many agree that it is a physician in clinical practice who applies pedagogy to their teaching and often participates in educational scholarship.^3^ Clinician educators constitute the majority of academic faculty members.^1^ At our highly research-intensive medical school, more than 60% of faculty are clinician educators.^4^ Clinicians choose an academic setting from a large pool of job opportunities because their joy comes from making a difference to patients, learners, colleagues, and health systems.^1, 5–8^ The clinical productivity contributed by clinician educators forms the financial foundation for academic medicine.^2, 9^ Of the academic activities supported by clinical revenue, the largest portion goes to research, followed by education.^10, 11^ Given this important role of clinician educator faculty, their retention and satisfaction are critical.^9, 11^

However, clinician educators are more likely to leave academics than clinician researchers.^12^ About half of clinical faculty leave their academic practice within 10 years, with an accompanying replacement cost of $115,000 to $286,000 per person .^12^ Despite the good intentions and sincere efforts of academic leaders, clinician educators often feel uneasy, unsupported, and underappreciated.^12–15^ Many factors contribute, including (1) lack of clarity about academic clinician educator career paths^1, 9, 16^; (2) a shortage of strong external institutional partnerships to fund career development or set tacit milestones (e.g., as the National Institutes of Health (NIH) does for researchers); and (3) few senior clinician educators as role models. Without a clear mental model of an academic clinician educator career trajectory, chairs and chiefs struggle to guide faculty. Even when resources for career development are available, leaders may have little guidance to know what investments are helpful and at which time points.

## A Framework to Guide Today’s Academic Clinician Educators

As senior clinician educators, researchers, and leaders at a research-intensive academic medical center, we present best practices from the literature and from our experiences with a variety of approaches from within our institution. We highlight generalizable concepts and sequences, rather than proscribed terminology and timing, since the latter can vary by specialty and institution. We focus on junior and mid-career academic clinician educators, since actions during these critical early years can launch a faculty member on a path of success. The contribution of this framework is in its synthesis of multiple decades of research with the current realities of academic medicine into actionable steps for leaders. The framework has four components. First, we compare the academic context for clinician researchers vs. clinician educators. Second, we define prototype career pathways of academic clinician educators. Third, we share a timeline of clinician educator early career milestones. Finally, we recommend systems that can be implemented by chairs and chiefs to support clinician educators at all stages of their careers and to bridge across the multiple missions of academic institutions.

### Comparison: Academic Clinician Researcher vs. Clinician Educator Careers

Many academic leaders are clinician researchers who achieved success within different individual, institutional, and external contexts than those that exist for clinician educators today^17^ (Table 1). The primary aim of the academic clinician researcher is to advance their field. They need to obtain and sustain independent research funding and publish high-impact peer-reviewed publications, in addition to making meaningful contributions as clinicians, mentors, and teachers.^18^ In contrast, the aims of academic clinician educators are varied and may include outstanding patient care, teaching, mentoring, and role modeling as measured by clinical outcomes, learner assessments, and dissemination of innovations in multiple venues.^7, 8^ Both groups possess strong internal motivation. The researcher leaves a legacy through scientific contributions while the clinician educator does so through hands-on impact on individuals and advancement of clinical care or medical education. Finally, the academic clinician researcher often has additional training via fellowships that include a focus on grant writing and research methodology, whereas the clinician educator usually hones their skills on the job, supplemented by episodic workshops.

**Table 1:** A Comparison of Today’s Academic Clinician Researcher vs. Academic Clinician Educator

Career context	Career characteristic	Academic clinician researcher	Academic clinician educator
Individual	Primary aim	Independent research expertise and funding in a focused area	Outstanding clinical and educational outcomes
	Career motivator	To add knowledge by applying the scientific method; to leave a legacy through published work	To heal and teach by applying both art and science; to leave a legacy through other people
	Relevant training	Most with fellowship training that includes research methodology, grant writing, and biostatistics	Some with additional training in teaching methodology or education research. Many participate in occasional workshops or conferences
Institutional	Salary support	Salary support from external sources (e.g., NIH) in highly competitive process; minor internal contribution for clinical work	Salary support from within the institution for clinical work; some from education or intramural grants
	Time allocation	Majority time devoted to research; a protected research time often mandated by career awards	Majority time devoted to clinical care and teaching; rare funded time for scholarship
	Promotion expectations	Clearly defined and often rigid promotion criteria focused on grants and publications	More flexible promotion criteria with a variety of ways to demonstrate impact
	Dissemination strategies	Peer-reviewed manuscripts of original research	Some peer-reviewed publications of programs, curricular innovations, reviews Non-peer-reviewed chapters, books
External	Career mentorship	Expected by external funders; occasionally funded mentor (e.g., K24); mutual benefit externally visible (e.g., shared publications)	Dependent on internal systems or individual initiative; often unfunded mentor; mutual benefit less defined or visible
	Collaboration culture	Expected high degree of both internal and external collaboration as research teams	Individual interpersonal interactions mostly internal to the program or institution

Different institutional contexts for these faculty groups drive their careers along different trajectories. The researcher competes for external funding, while the clinician educator is funded through institutional clinical revenue.^1, 4, 11^ While both feel the increasing pressure of clinical revenue generation, research career development awards require on average 70% effort devoted to research.^19^ In contrast, there are few funding mechanisms for clinician educator scholarship.^20, 21^ One study reported that less than 30% of medical education research publications were associated with funding.^22^ Therefore, most clinician educators start their careers with approximately 75% effort devoted to clinical care.^1, 9, 23^ Clinical documentation may spill over into evenings and weekends, co-opting personal time for work. As a result, little time remains for academic pursuits and scholarships, which are often required for obtaining leadership roles and academic promotion.^23, 24^

A final difference between the two faculty groups lies in the mentorship and collaboration structures. Chew reported that a clinician researcher faculty member was 10 times more likely to have a mentor than a clinician educator.^25^ Mentorship is fundamentally built into the structure of the researcher’s career and is often required by external stakeholders, such as NIH. An essential component of a funded mentored research career development award is strong mentor support, a relationship that generates an individualized career development plan for the junior clinician researcher’s success. In contrast, few clinician educator roles include a requirement to work with a mentor on a career development plan. Furthermore, the clinician researcher path includes an expectation for robust internal and, over time, external collaborations. This is less true for clinician educators, who are deeply knowledgeable about their own institution, but find external collaboration hampered by unique characteristics of every institution. Without a robust body of academic products that result from these working relationships, clinician educators risk respect and promotion at some institutions.^26–29^

### Pathways: Career Prototypes for Academic Clinician Educators and Strategies for Leaders

Enhancing mentorship and collaboration for clinician educators requires understanding their career prototypes and associated milestones. We update Levinson’s 1998 models and propose four prototypical careers in two categories to capture the essence of most academic clinician educator careers today ^1^ (Table 2). While there is a continuum, mentors should start by asking these “dual professionals” whether they see themselves as *clinician* educators who devote the majority of their attention to patients and health systems (“BIG C, little e”), or clinician *educators* who devote the majority of their energy to education through curriculum development, leadership, assessment, and/or scholarship (“little c, BIG E”).^1, 30^

**Table 2:** Four Protype Career Pathways for Academic Clinician Educators

Career pathways	Sample roles	Time allocation	Scholarship possibilities	Recommended support from chiefs, chairs, and mentors
“BIG C” *clinician* educators				
Master clinician	Generalist, hospitalist, or subspecialist focused on clinical mastery	Majority time in patient care, often includes clinical teaching or mentoring	Case reports, clinical reviews, book chapters, grand rounds	Allocated time for reading and scholarship; workshops on teaching and feedback; funds to participate in national professional societies, nomination for local or national master clinician awards.
Clinician leader	Medical director of ambulatory or inpatient service	Most time in clinical care and clinical program leadership, includes some clinical teaching or mentoring	Program development and evaluation, clinical guidelines, quality improvement, policy statements	Allocated time and guidance for scholarship; training in leadership, quality improvement, health care financing; funds to participate in national professional societies; nomination for local or national committee or leadership positions
“BIG E” clinician *educators*				
Education leader	Director of course, clerkship, residency, or fellowship program	Variable time allocation to patient care and education leadership responsibilities	Curricular innovation, program evaluation, education-related manuscripts (e.g., trainee wellness, diversity, or professionalism)	Allocated time and guidance for scholarship; training in curriculum development, learner assessment, program evaluation; encouragement and funds to present at education national meetings; nomination for local or national education positions
Education researcher	A clinician educator with additional training (e.g., master’s or doctoral degree)	Variable time allocation to patient care and education research, mentorship, or education journal editorships	Education research, education perspectives, education review articles	Allocated time for research; funds for training in education research methods; encouragement to collaborate and mentor within and across institutions; support for intramural education research grants and awards

#### “BIG C” **Clinician** Educators

“BIG C” clinicians sort into two types: the master clinician and the clinician leader. Some move through both in sequence over the span of a career. The budding master clinician is a high-volume clinical expert who may also teach and mentor. Master clinicians are seen as the doctor’s doctor, yet other than patient satisfaction scores, this accomplishment may be difficult to translate to a regional, national, or international reputation important at some institutions. Other “BIG C” faculty aspire to lead clinical programs, divisions, or departments. Their academic and clinical success is measured by their role in program design and administration, quality improvement, and policy development. Chairs and chiefs can support “BIG C” clinicians by helping them allocate time for reading, offering funds to attend professional conferences or other trainings to hone their clinical and leadership skills, sponsoring them for national committee roles, and nominating them for awards or positions.

#### “BIG E” Clinician **Educators**

Aspiring “BIG E” educators aim to become education leaders and/or education scholars. The education leader directs a medical student course, clerkship, residency, or fellowship program.^1^ Relevant scholarship includes development and dissemination of curricular innovations, program evaluations, or studies of teaching outcomes. This faculty member partners with education researchers to conduct qualitative or quantitative education research.^9^ Chairs and chiefs can support “BIG E” educators by encouraging them to pursue additional education skills training, apply for internal or external innovation or education grants, establish a partnership with education researchers, and disseminate their work. Leaders can sponsor them for national academic committees and nominate them for awards or positions.

### Milestones: a Sample Timeline for Leaders to Guide Early Academic Clinician Educators 

#### Years 1-3 for All Clinician Educators: Build Clinical Skills, Identify Niche, Refine Teaching

**Figure 1:**
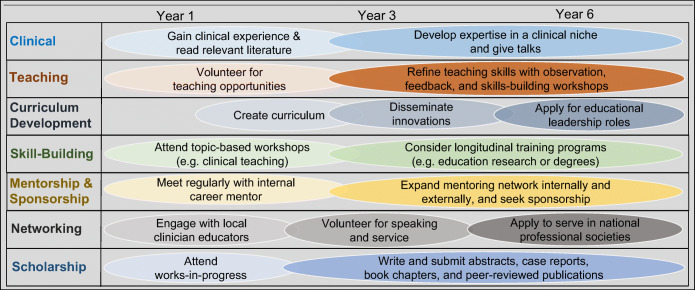
Early and mid-career milestones for academic clinician educators in multiple domains.

Sequential career milestones can guide all clinician educators early in their careers (Fig. 1). Starting a career as a strong clinician is important to build credibility and serves as fertile ground to find one’s area of interest.^3, 9, 31^ Early on, clinician educators begin to define an area of interest, or a niche, and build a targeted knowledge base.^32^ Identifying an academic niche starts with an unanswered question, an imperfect system, or a topic that sparks curiosity. Sample niche areas include a specific medical condition, the clinical reasoning curriculum, or a learner assessment strategy. Chairs and chiefs can advise all clinician educators to develop solid competence as a clinician and find a niche, regardless of future career pathways.

In parallel, junior clinician educators will gain teaching experience and skills.^8, 32^ Exploring volunteer teaching opportunities allows for recognition of one’s own passion for settings (e.g., classroom or clinical) and learners (e.g., students or residents).^1, 31^ In addition, this helps the clinician educator build a reputation for excellence within close-knit local education communities. Skills-building can occur in many ways, from workshops to longitudinal competitive programs, such as the well-known Macy Foundation programs.^3, 31, 33, 34^ Important education competencies include teaching and feedback, support for diversity, equity and inclusion, curriculum development, and leadership skills.^3^ Many faculty members aim for funded education positions, but almost all start with volunteer teaching and skills-building. Chairs and chiefs can advise early clinician educators to volunteer for teaching opportunities and attend education skills workshops.

#### Years 4-6 by Career Prototype: Develop Niche, Build Networks, Create Scholarship

After a few years, clinician educators should be able to develop a niche, a path, and a network.^35, 36^ For “BIG C” clinicians, a network may consist of professional communities focused on a medical condition (e.g., HIV) or a clinical setting (e.g., home care).^32^ Among “BIG E” educators, focus on a learner level (e.g., medical students), or an instructional format (e.g., simulation), is common.^1^ As they build expertise, junior faculty will further grow their network by delivering talks and participating in academic societies. Grand rounds are excellent ways to reveal honed expertise to senior colleagues who may share grant, publication, or leadership opportunities. Chairs and chiefs can guide clinician educators to develop and declare their niche and strengthen their ties to professional communities.^37^

As promotion to associate professor approaches, clinician educators will need to demonstrate scholarship and dissemination.^27, 25, 38, 39^ At some institutions, it is necessary to demonstrate a national reputation, often through invitations for talks at conferences or other institutions or via high-impact publications. Clinician educators are less likely to have time allocated to scholarship built-in to funded time, and yet scholarly productivity is expected of all faculty for advancement at research-intensive institutions.^1, 28, 40, 41^ Once chairs and chiefs apply the framework of different prototypes and milestones, they can design systems to support scholarship and success along each career path. For example, budding master clinicians should be encouraged to give presentations, write reviews, or co-author book chapters. Clinician administrators can describe quality improvement efforts or clinical practice guidelines and submit abstracts or workshops to local, regional, or national conferences. The education program director should submit curricular innovations for peer review (e.g., MedEdPORTAL). Education researchers can collaborate within and across institutions to address unanswered questions in medical education.^36, 39^

### Systems: Supporting Clinician Educators by Career Stage

Chairs and chiefs at all academic institutions can design systems to support clinician educators at all stages of their careers (Box). While institutions have different leadership structures, these recommendations apply to division chiefs and department chairs with direct supervisory and budgetary responsibility over clinician educator faculty. Some institutions have piloted an education value units system.^42–44^ The strategies described here are more comprehensive and center on mentorship, sponsorship, community, and infrastructure. When applied, they have the potential to enhance job satisfaction, strengthen community, increase scholarly output, improve success in advancement, and amplify the visibility and reputation of the institution.

**Box.** A Checklist for chairs, chiefs, and mentors in supporting clinician educators

**Table:** 

**For All Clinician Educators:**	
*Community Building*	
□ Include the clinical and education missions in all activities and acknowledgements (e.g. education-focused grand rounds, newsletter celebrating successes) ^*,†^	
□ Invite all unit faculty (including clinician researchers) to participate in focused high-priority clinical and teaching activities and discussions ^*,†,‡^	
□ Require all senior faculty members to sponsor others by inviting junior clinician educators to co-author case reports, invited commentaries, or book chapters ^*,†,‡^	
□ Encourage participation on local committees and national professional societies ^*,†,‡^	
□ Introduce clinician educators to colleagues locally and nationally ^*,†,‡^	
*Resource Allocation*	
□ Designate a 10-20% funded associate chair^*^ or chief^†^ to oversee clinician educators’ advancement, wellness, and support	
□ Fund 10-20% start-up academic time for 1-2 years to launch new clinician educators ^*,†^	
□ Consider funding a 6-month scholarship sabbatical for mid-career clinician educators ^*,†^	
□ Provide funds for faculty development (e.g., writing workshops), career expenses (e.g., meeting registration), and administrative tasks (e.g., scheduling assistance) ^*,†,‡^	
□ Nurture philanthropic support for clinical programs, education programs, and clinician educator faculty members (e.g., endowed chairs) ^*,†^	
**For Junior Clinician Educators:**	
□ Match new clinician educators with clinician educator mentors and encourage additional internal and external organic mentor pairings ^*,†^	
□ Structure mentoring around individual development plans ^*,†,‡^	
□ Encourage all clinician educators to identify a clinical and/or education niche ^*,†,‡^	
□ Enhance skills with workshops and works-in-progress sessions ^*,†,‡^	
□ Institute a pre-promotion CV review meeting (during years 3 or 4) to identify gaps ^*,†,‡^	
**For Mid-Career Clinician Educators:**	
□ Review mentor assignment; change (as needed) and expand their mentor network ^*,†^	
□ Nominate for awards, speaking opportunities, and/or leadership positions ^*,†,‡^	
□ Include clinician educators in grant applications (e.g., to co-lead patient education) ^*,†,‡^	
□ Encourage a writing habit and aim for at least 1-2 publications per year ^*,†,‡^	
**For Later-Career Clinician Educators:**	
□ Encourage additional training, and serving as a trainer, in mentorship and sponsorship^*,†^	
□ Match with junior clinician educators as career mentors^*,†^	
□ Facilitate discussions about legacy and succession of educational leadership positions^*,†^	
□ Identify funding mechanisms to support as mentor, sponsor, scholar, and/or leader^*,†^	

In the text box, the superscripts indicate:

*Actions recommended for department chairs

†Actions recommended for division chiefs

‡Actions recommended for mentors

#### All Clinician Educators

All faculty benefit from supportive communities for satisfaction and advancement.^35, 45^ Local and national peer mentorship communities enable clinician educators to discuss best practices, share resources, promote equity, and prevent burnout.^41, 46, 47^ Local medical educator academies can support educator development and fund education innovations. Membership in educator academies can signal achievement and open doors to tangible support.^48, 49^ Within a division, leaders can include clinician educators along with research faculty in works-in-progress discussions, celebrations of teaching awards and successful curricula, and sharing of opportunities. Chairs and chiefs play a critical role in breaking down silos and creating supportive and inclusive academic communities across missions and institutions.

National professional societies can also serve as important hubs for clinician educators. They facilitate external networking and allow faculty to benefit from society initiatives. In these communities of practice, faculty from different institutions can share innovations and challenges, engage in collaborative scholarship, and connect with external promotion referees. Some societies directly support clinician educators with financial resources for meetings or grants.^50–52^ Others integrate faculty development on medical education topics into annual meetings and create venues to present medical education scholarship, as well as clinician educator mentoring programs.^53–56^ Chairs and chiefs can encourage, fund, and release clinician educator faculty to attend regional and national professional society meetings.

Chairs and chiefs should invest in supporting clinician educators.^1, 11^ For example, a senior clinician educator could be appointed into a funded position as associate chair or chief to partner in creating support systems. Also, leaders could fund start-up academic time for new clinician educators, scholarship sabbatical months for mid-career clinician educators, and release from clinical revenue generation for attending longitudinal training programs. These relatively small expenditures can make a difference in catalyzing academic success, sustaining wellness, and promoting retention. Likewise, infrastructure for decreasing administrative tasks for clinician educators can increase efficiency and output. Examples include assistance with scheduling, reimbursement, documentation, billing, or manuscript preparation. Some of these costs can be reallocated from excess clinical revenue, either within a division for those with higher reimbursement or by applying a funds flow system that crosses different subspecialties. Another strategy is to nurture philanthropic support for the clinical and education missions.^11^ New chairs and chiefs could negotiate for funds to support clinician educators in their recruitment package. In summary, chiefs and chairs have a number of options available to provide tangible financial support and protected time for clinician educators at all stages of their careers.

It is important to highlight that gender disparities persist in academic medicine.^57–59^ Advancement and compensation gaps for women are significant and widen with seniority.^57, 58^ The early career years coincide with family building for many faculty, and women may shoulder outsized responsibilities, spend more time providing dependent care, and receive less institutional support (e.g., research funding or administrative assistance).^60^ To support women faculty, chairs and chiefs can ensure equal compensation, decrease after-hour meetings or work, offer a fair part-time track, provide support to help maintain productivity, prioritize mentorship and sponsorship, improve search and promotion committee practices, require implicit bias training, and oppose discrimination or harassment.^60–64^ Family-friendly programs and administrative support can help.^60, 61^ Not surprisingly, success has been reported with targeted protected time to allow immersion into activities that lead to professional growth and leadership roles.^62, 65, 52^ Interventions on multiple levels (i.e., individual, interpersonal, institutional, community, and policy) are necessary to recruit, retain, and advance the careers of women clinician educator faculty in academic medicine.^61, 62^

Similarly, faculty who are underrepresented in medicine (URM) face significant obstacles. Proportionally, there are fewer URM faculty in academic medicine than the general population, particularly at the highest levels (e.g., professor, chair, and dean).^66^ Minority faculty report lower career satisfaction, an increased sense of isolation, lack of mentoring, higher debt burden, and higher likelihood of leaving academics.^67–69^ Chairs and chiefs should work to eliminate disparities in academic processes and extra responsibilities placed on URM faculty (i.e., the minority tax).^67, 70, 71^ Diversity efforts need to count in advancement.^71^ Subtle negative impacts on academic advancement of providing clinical care to the underserved should be addressed.^71^ Racism, stereotyping, bias, and discrimination in recruitment and promotion must be eradicated.^71^ URM academic mentoring groups and faculty development curricula can promote networking, skills-building, a sense of belonging, and engagement of senior faculty as career sponsors.^71–77^ Supporting these mentoring groups with a small amount of monies and administrative help is an effective use of funds. At some institutions, supplemental salary support for URM faculty advances equity.^74, 76^ In summary, leaders have many tools to improve the diversity climate for URM clinician educator faculty.

#### Junior Clinician Educators

For junior clinician educators, mentorship is critical.^35, 78^ A mentored clinician educator spends twice as much time in scholarly activity compared with non-mentored clinician educators.^25^ Lack of mentorship is associated with fewer peer-reviewed publications.^29^ Clinician educators can be matched with a mentor from the start, with a goal to expand to a team of mentors to support the faculty member’s area of interest.^35^ The mentor uses an individual development plan at mentoring meetings and partners with the chair and chief in guiding the junior clinician educator.^79^ While exploring opportunities, the mentor can help distinguish stepping-stones from distractors and cultivate an awareness of what brings joy. If there are no senior clinician educators to serve as mentors, faculty from other departments can be recruited in exchange of nominal support and recognition. To start, chiefs and chairs can begin by pairing all new clinician educators with at least one senior clinician educator mentor, and meeting with each to decide whether a good match has been made**.**

#### Mid-Career Clinician Educators

For mid-career clinician educators, sponsorship from chairs, chiefs, and other senior faculty members can catapult a career onto the national stage.^80–82^ Leaders can introduce faculty to colleagues for committee membership or professional collaborations such as multi-institutional research projects, presentations, and workshops. If a network locally and nationally does not already exist, the chair and chief could make connections. Research colleagues can be encouraged to include clinician educators in grant applications or invited manuscripts, and vice versa. Eventually, clinician educators can be recommended for speaking opportunities, achievement awards, and leadership positions. Chairs and chiefs are influential in opening doors, and mid-career clinician educators can benefit in particular from this type of sponsorship.

#### Later-Career Clinician Educators

Clinician educators are apt to examine their careers over time, and chairs and chiefs can support this process.^83^ To leverage their expertise, senior clinician educators could both refresh their own mentorship and sponsorship skills and train others to be effective mentors and sponsors for junior clinician educators. Experienced clinician educators may consider adding junior and mid-career faculty members to their contracts, grants, and writing teams. Many senior clinician educators will choose to turn over clinical or education leadership positions at 5, 10, or 15 years to promote legacy planning and allow for exploration of other interests (e.g., writing). Open conversations between leaders and later-career clinician educators can help them to maintain satisfaction in their own careers while leveraging their expertise to support rising junior clinician educators and leaders.

## Future Directions

This proposed framework is offered by experienced faculty at one research-intensive institution to advance debate on how to support academic clinician educator careers. Thriving clinician educators are crucial for flourishing academic medical centers with the triple mission of discovery, education, and patient care. Research is needed on clinician educator work experience, engagement, and retention. At many institutions, promotion and tenure policies can be further tailored for clinician educators. Models of funding career advancement need to be piloted and, if successful, disseminated. Partners, such as the American Medical Association and the Association of American Medical Colleges, should consider their role in advancing clinician educator careers. These suggestions can be tested at different institutions with varying focus on each of the three missions. Producing the next generation of physicians is an essential task; faculty members who undertake this role should be supported and advanced.

## Data Availability

authors have no relevant conflicts of interest
